# Understanding relations between intolerance of uncertainty and body checking and body avoiding in anorexia nervosa

**DOI:** 10.1186/s40337-022-00647-1

**Published:** 2022-08-18

**Authors:** Jojanneke M. Bijsterbosch, Anouk Keizer, Paul A. Boelen, Femke van den Brink, Lot C. Sternheim

**Affiliations:** 1 Department of Clinical Psychology, Utrecht University, PO Box 80140, 3508 TC Utrecht, The Netherlands; 2 Department of Experimental Psychology, Utrecht University, Utrecht, The Netherlands; 3 ARQ National Psychotrauma Centre, Nienoord 5, 1112 XE Diemen, The Netherlands; 4 ARQ Centrum’45, Nienoord 5, 1112 XE Diemen, The Netherlands

**Keywords:** Anorexia nervosa, Recovered, Body checking, Body avoiding, Intolerance of uncertainty

## Abstract

**Background:**

A key feature of anorexia nervosa is body image disturbances and is often expressed by dysfunctional body-related behaviours such as body checking and body avoiding. These behaviours are thought to contribute to both the maintenance and relapse of AN, yet empirical evidence is scarce. One variable that may contribute to the need for engaging in these behaviours is intolerance of uncertainty. This study aims to investigate body checking and body avoiding and its relations with intolerance of uncertainty in women with anorexia nervosa (AN-ill; 70), women recovered from AN (AN-rec; 85) and control group (127).

**Methods:**

Three questionnaires were completed, measuring eating pathology, intolerance of uncertainty and body checking and body avoiding. One-way ANOVAS were used to test group differences. Moderation analyses were used to investigate associations between variables.

**Results:**

Levels of intolerance of uncertainty, body checking and body avoiding were highest in AN-ill followed by AN-rec and, lastly, the control group, confirming group differences. Intolerance of uncertainty was associated with body checking in the AN-rec group and the control group but not in the AN-ill group. The association between intolerance of uncertainty and body avoiding was reported in the AN-rec group and only marginally in the control group.

**Conclusion:**

Levels of intolerance of uncertainty, body checking and body avoiding were highest in AN-ill, however still elevated in AN-rec, confirming the presence of body image disturbances, even after recovery. The unique associations between intolerance of uncertainty and body checking and body avoiding within the studied groups may represent different stages of the illness. In the AN-rec group, the relation between intolerance of uncertainty, body checking and body avoiding may be driven by trait anxiety. For AN-ill group, body checking and body avoiding may eventually have grown into habitual patterns, rather than a strategy to ameliorate anxiety and uncertainty.

**Plain English summary:**

Women with anorexia nervosa often experience disturbances in their body image and are expressed in body-related behaviours such as body checking and body avoiding. These behaviours are thought to contribute to both the maintenance and relapse of anorexia nervosa. Intolerance of uncertainty is defined as the incapacity to tolerate uncertainty and may contribute to the need for engaging in these behaviours. This study aims to investigate body checking and body avoiding and its relations with intolerance of uncertainty in women with anorexia nervosa (AN-ill; 70), women recovered from AN (AN-rec; 85) and control group (127). Three questionnaires were completed, measuring eating pathology, intolerance of uncertainty and body checking and body avoiding. Levels of intolerance of uncertainty, body checking and body avoiding were highest in AN-ill and still elevated in AN-rec, even after recovery. The associations between intolerance of uncertainty and body checking and body avoiding within the studied groups may represent different stages of the illness. In the AN-rec group, the relation between intolerance of uncertainty, body checking and body avoiding may be driven by trait anxiety. For AN-ill group, body checking and body avoiding may have grown into habitual patterns, rather than a strategy to ameliorate anxiety and uncertainty.

## Introduction

Anorexia nervosa (AN) is a severe and disabling eating disorder that carries a high disease burden for affected individuals, their loved ones, and society [55]. Treatment outcomes are poor: 20% of patients remain chronically ill and less than 50% reach full recovery [57]. Key diagnostic features of AN include disturbances in the way in which one’s body is experienced and excessive influence of body weight and shape on self-evaluation (APA [2]). Body image disturbance symptoms may consist of perceptual deficits, cognitive-affective distortions, and dysfunctional body-related behaviours such as body checking and body avoiding (e.g., [38].

Specifically, over the past two decades increased attention has been given to body checking and body avoiding, which have been identified as potential etiological and maintenance factors in eating disorder pathology (e.g., [24, 64]). Furthermore, research has found that individuals who are recovered from AN still show a partially disturbed body image [20], 22. However, studies on body checking and body avoiding in women recovered from AN are scarce [4]. Consequently, it is unclear to what degree body checking and body avoiding persists in women recovered from AN and whether these behaviours might potentially be implicated in relapse. Additionally, there is currently little understanding of potential mechanisms underlying the behavioural component of body image disturbances. A deeper understanding of these dysfunctional behaviours, and their underlying mechanisms in individuals with AN and recovered from AN is important as it may directly inform treatment interventions for AN.

Body checking is defined as frequent and repetitive behaviour in which an individual monitors his or her body in multiple ways [38, 45] through behaviours such as ritualistic weighing, compulsive mirror checking, and using the fit of clothes to judge weight changes (e.g., [45]. Specifically, body checking behaviours, which are often brief but repeated frequently, may result in amplifying the perceived imperfections in body shape or weight that commonly contribute to body dissatisfaction in AN ([23], 25. Although levels of body checking might clearly distinguish women with and without AN, findings from studies in nonclinical samples of women suggest that body checking is positively associated with greater eating disorder symptom severity [45, 50, 65] and thus should be regarded as a risk factor within this group as well [4, 11, 53]. Regarding women recovered from AN, there is only one study, in which Bamford and colleagues (2014) found no differences in levels of body checking in weight restored women versus low weight women. This suggests that body checking remains high even when individuals restore their weights during recovery, however, these results require further replication.

A concept that is closely related to body checking is body avoiding [53]. Body avoiding is characterized by efforts to avoid seeing one’s body weight and shape and includes behaviours such as covering mirrors, wearing oversized clothes and refusing to be weighed [24, 51]. Relative to body checking, body avoiding tends to be less overt and more difficult to detect [65]. Consequently, relatively little is known about body avoiding in AN. This lack of understanding is concerning as some researchers have theorized body avoiding behaviours are indicative of more severe eating disorder pathology compared to body checking [24]. Indeed, a handful of studies on body avoiding confirm that women with AN showed greater body avoiding when compared to the control group [39]. In a group of individuals with AN, women with low weight reported higher levels of body avoiding relative to women whose weight was restored. Additionally, it was found that levels of body avoiding were lowest in the control group [4]. However, further evidence suggesting body avoiding may be a risk factor for developing eating disorder symptoms. An experimental study in college students found that avoidance of body and shape information led to increased disordered eating attitudes [21].

These behavioural manifestations of overevaluation of weight and shape have been theorized to hold an anxiolytic function [44, 49, 65]. That is, engaging in body checking and body avoiding may serve to reduce the high anxiety that is inherent to AN [34, 47]. In other anxiety-related psychological disorders (e.g., obsessive compulsion disorder), checking behaviour has been conceptualized as a behaviour shown in situations characterized by negative affect (e.g., anxiety), and performed to reduce this negative affect or avoid feared outcomes or events (Hartmann et al. [30]). Maladaptive avoidance, on the other hand, is broadly viewed as any attempt to downregulate unpleasant experiences through avoidance, escape, suppression, distraction, or control (e.g., social anxiety disorder; [15]. Ultimately, this particular behaviour can lead to impairment because it prevents disconfirming experiences and insulates the individual from learning about the actual outcomes of one’s behaviour [18]. Research has linked both avoidance and checking behaviour to anxiety disorders [5, 19]. Eating disorders are characterized by high levels of anxiety [40], with anxiety disorders co-occurring within 80% of individuals with a diagnosis of an eating disorder [47].

Indeed, cognitive behavioural models of eating disorders suggest that body checking and body avoiding may serve as a safety behaviour, reducing anxiety and distress in the short-term [25] but worsening body image, negative affect, and eating-related cognitions and behaviours in the long run [3, 44]. Two experimental studies found that body checking led to a decrease of either negative emotions [67] or negative affect [64] *after* the checking period, in comparison to levels of emotions and affect prior to the checking episode. Both studies were conducted within a community sample of women. Wilhelm et al. [67] suggested that this finding confirms that, at least in the short run, body checking decreases negative emotions. Nevertheless, the precise function of body checking and body avoiding and its association with anxiety in both clinical and nonclinical groups is not fully understood yet [44], 64. Anxiety may both precede and follow episodes of body checking and body avoiding [64]. Regarding AN in particular, the expression of the behavioural component of body image might function differently across stages of illness, specifically seeing the impact of low weight and related emotional states.

One key anxiety-related cognitive process that may be contributing to body checking and body avoiding is intolerance of uncertainty (IU) and has been identified as transdiagnostic factor underpinning anxiety and anxiety driven behaviours [9], 12, 36, 58 and has been defined as a tendency to have negative perceptions and reactions to ambiguous stimuli [13]. Individuals with higher levels of IU experience anxiety and negative affect in uncertain situations even when the potential threat is minimal. Higher levels of IU may intensify the threat meaning assigned to an object or situation (i.e., the body). Reactions to IU include obsessions, ritualized behaviours, compulsions and avoidance, which are utilized to reduce uncertainty and anxiety [9]. These possible reactions are strikingly similar to body checking and body avoiding. Body checking might be used as a method to obsessively gather as much information as possible on the body (e.g., weighing oneself), aiming to obtain satisfactory levels of certainty about their weigh and shape. Body avoiding (e.g., refrain from looking in a mirror) might stem from the paralyzing effect dealing with uncertainty about the body may cause.

Several studies reported significantly higher degrees of IU in AN, relative to the control group, as well as strong associations between higher IU and more severe eating disorder symptoms [10, 27, 59]. Only a few studies examined IU in women recovered from AN [41, 46, 48], with all studies reporting less severe IU scores in women recovered from AN compared to symptomatic patients. A handful of studies have recently linked IU to body image disturbances in clinical groups [7, 10, 27] and nonclinical groups [8]. From a theoretical perspective, establishing this link is essential as it will inform the development of conceptual models on the role of IU in both acute AN and recovered AN. From a clinical perspective, it is important to establish this relation as it might improve the effectiveness of early intervention programs and contribute to the development of appropriate (relapse) interventions.

### The present study

Firstly, this study aimed to examine the reported levels of body checking and body avoiding in women with AN (AN-ill), women recovered from AN (AN-rec), and without AN (control group). Regarding levels of body checking, it was expected that reported levels were highest in the AN-ill and AN-rec group [4], and lowest in the control group. Levels of body avoiding were hypothesized to be highest in the AN-ill group, followed by AN-rec and, lastly, by the control group [4, 38]. Secondly, this study aimed to investigate levels of IU in AN-ill, AN-rec and the control group. It was hypothesized that AN-ill would report the highest levels of IU, followed by the AN-rec and subsequently by the control group [10, 41, 48].

Finally, we examined the associations between IU and body checking and body avoiding. It was hypothesized that the relations between IU and body checking and body avoiding were moderated by group. Specifically, it was expected to find the strongest association in the AN-ill group, followed by the AN-rec and the control group.

## Methods

### Participants

Data were collected via a survey in Qualtrics distributed on various international online platforms, such as eating disorder support groups, survey sharing pages, and other social media. The survey was in English and data collection was exclusively based on self-report. Inclusion criteria were female and a minimum age of 18 years. Respondents were asked to provide informed consent prior to starting the survey. This research was approved by the Utrecht University Faculty Ethics Review Board (FETC; #19–056).

At the start, a total of 492 individuals participated in the present study. Participants were categorized as current AN-ill, AN-rec, or in the control group, based on their self-reported eating disorder diagnosis (AN-ill; AN-rec; no history of eating disorder) and their Eating Disorder Examination Questionnaire (EDE-Q) scores (see e.g., Slof-Op ‘t Landt et al. [54]). Following the criteria proposed by Bardone-Cone et al. [6], participants were assessed on their physical, behavioural and cognitive status. Based on the criteria proposed by Bardone-Cone et al. [6], 282 participants ultimately met these strict criteria and could be divided into the three groups. All other participants were excluded from the study.

For the *physical status*, in the AN-ill group, a Body Mass Index (BMI) ≤ 18.5 kg/m^2^ was required. For the AN-rec group a BMI between 18.5 and 24.9 kg/m^2^ was required. For the control group, a BMI of between 18.5 and 24.9 kg/m^2^ was required. We used self-reported weight and height.

*Cognitive recovery* The Eating Disorder Examination Questionnaire (EDE-Q) version 6.0 was used [23]. It provides a coverage of eating disorder cognitions over the past 28 days: restraint, eating concern, weight concern, shape concern. In this study, an EDE-Q global scale was calculated (22 items; range 0–6). Scores within 1 SD of age-matched community norms [42] were required for either cognitive recovery or the absence of eating disorder cognitions. In a large Dutch sample, it was previously shown that the EDE-Q global score was highly accurate in discriminating between individuals with an ED and those without [1]. In this study, Cronbach alpha’s for global score was 0.97.

*Behavioural recovery* was assessed using questions that were based on the open questions of the EDE-Q and were referring to either the presence or absence eating disorder behaviours such as binge eating, vomiting, laxative use and fasting. An example of such a question was: *Do you ever make yourself sick because you feel uncomfortably full?* Women in both the AN-rec the control group reported the absence of these eating disorder behaviours. Thus, to be included in either the AN-rec group or in the control group, the required answer to either of these questions had to be “*no*” and when included in the AN-ill group one of the answers to these questions was required to be “*yes*”.

## Measures

### Body checking and body avoidance

The Body Checking and Avoidance Questionnaire (BCAQ; [38] is a 30-item questionnaire to measure eating-disorder-related psychopathological forms of body checking, body avoidance, and reassurance seeking behaviours on three subscales. An example of the body checking subscale is “I test whether I can reach around my wrists and ankles with one hand.” An example of the body avoiding subscales is “I don’t wear any clothing which show my feminine curves, e.g., jeans or tight tops.” Items can be answered on a 4-point Likert scale ranging from 1 (*not at all true*) to 4 (*very true*). In the present study, the BCAQ had an excellent internal consistency (α = 0.96), the body checking subscale was (α = 0.94) and the body avoiding subscale was (α = 0.93).

#### Intolerance of uncertainty

The Intolerance of Uncertainty Scale (IUS-12; [13] measures one’s IU as expressed in several domains, including emotion, cognition and behaviour. The IUS-12 consists of twelve items, scored on a 5-point Likert scale ranging from 1 (*not at all characteristic for me*) to 5 (*entirely characteristic for me*). behaviour. It has two subscales, including prospective IU, which measures cognitive distress, and inhibitory IU, which measures behavioural inhibition. An example of an item of the prospective IU subscale is “It frustrates me not having all the information I need.” An example of the inhibitory IU subscale is “When it is time to act, uncertainty paralyses me”. Scores were calculated by summing up the respective items, with higher scores indicating higher levels of IU. In the current study, the IUS-12 total had an excellent internal consistency (α = 0.94).

## Statistical analyses

For analysing the data IBM SPSS Statistics version 26 [33] was used. A power calculation was carried out, using G*Power [26]. This sample size calculation indicated that based on a medium effect size the sample size was large enough to reliably execute the statistical analyses [32]. Firstly, groups were compared regarding IU (IUS-12), body checking and body avoiding (BCAQ) with analyses of variance. Significant group differences were followed up with planned contrast analyses. To explore associations between IU, body checking and body avoiding, moderation analyses were conducted using PROCESS [31]. Specifically, linear regression analyses were calculated using IU as an independent variable, body checking and body avoiding as dependent variables, and group (1 = control group, 2 = AN-rec and 3 = AN-ill) as multicategorical moderator variable. Indicator coding was used for these three groups [43]. Separate models were run for body checking and body avoiding, two moderation models were tested in total. The alpha level was set at *p* < 0.05 and *p*-values between 0.05 and 0.10 are denoted as marginally significant.

## Results

### Participants

A total of 282 individuals participated in this study. Seventy participants (Age = 24.5; *SD* = 5.90) self-reported a primary AN diagnosis according to the DSM 5 (APA [2]), had a BMI below 18.5 (BMI = 16.5; range = 11.1–18.6; *SD* = 1.8), their score on the global EDE-Q was above the clinical cut off (global EDE-Q = 4.8; range = 2.6–6.0; *SD* = 0.82; [1] and confirmed the presence eating disorder behaviours such as binge eating, vomiting, laxative use and fasting.

The AN-rec group consisted of 85 participants (24.9; *SD* = 4.09) who identified themselves as fully recovered. Their BMI was between 18.5 and 24.9 (BMI = 21.07; range = 18.6–25.4; *SD* = 1.86), their score on the global EDE-Q was below the clinical cut off (EDE-Q = 1.18; range = 0.00–3.27; *SD* = 0.80; [1] and they confirmed the absence of eating disorder behaviours.

The control group consisted of 127 participants (age = 22.0; *SD* = 1.91) who did not report a prior or a current AN diagnosis. Their BMI was between 18.5 and 24.9 (BMI = 21.57; 18.51–24.92; *SD* = 1.55), their score on the global EDE-Q was below the clinical cut off (EDE-Q = 87; range = 0.00–2.45; *SD* = 0.65; [1] and they confirmed the absence of eating disorder behaviours.

### Descriptive statistics

Table 1 displays the means and the standard deviations of the study’s variables across groups. The levels of IU found in the AN-ill group of this study were higher than found in a study by Sternheim et al. [60]. Regarding the levels of IU found in the control group were lower than reported in the same study by Sternheim et al. [60]. Levels of IU, measured with the IUS 12, have not been reported yet.

**Table 1:** Means and standard deviations of questionnaire measures as function of group (N = 282)

	AN-ill			AN-rec			Control group					
	*n*	*M*	*(SD)*	*n*	*M*	*(SD)*	*n*	*M*	*(SD)*	*F*	*p*	$\eta_{p}^{2}$
Total IU	70	45.24^a^	(10.80)	85	32.74^b^	(10.49)	127	23.36^c^	(7.72)	122.40	< .001	.467
BC	70	2.84^a^	(.72)	85	1.71^b^	(.51)	127	1.45^c^	(.32)	179.31	< .001	.562
BA	70	2.93^a^	(.73)	85	1.81^b^	(.63)	127	1.24^c^	(.23)	231.55	< .001	.624

*BA* Body avoiding, *BC* Body checking, *IU* Intolerance of uncertainty

Means with different superscript are significantly different from each other (a > b > c)

Within the AN-ill group, levels of body checking obtained in this study were similar to the levels obtained in a study by Legenbauer et al. [38], using the BCAQ. However, levels of body avoiding were higher relative to that study. Within the control group, levels of body checking were slightly higher than in in that same study. This contrasts with the levels of body avoiding which were lower than the reported levels in Legenbauer [38]. Levels of body checking and body avoiding, measured with the BCAQ, have not been reported yet.

### Groups comparisons

#### Body checking

Partly in accordance with our hypothesis, groups significantly differed in levels of body checking with a large effect size (Table 1). It was expected that the reported levels of body checking were highest in the AN-ill and AN-rec group [4], and lowest in the control group, we performed two planned contrast analyses for body checking. Firstly, we found that both AN-ill, and AN-rec were significantly different from the control group (t (187.126) = 14.125, *p* < 0.001). Secondly, the planned contrast analysis for body checking showed a significant result between all three groups as well (t (83.788) = 15.306, *p* < 0.001) and confirmed group differences. Reported levels of body checking were highest in the AN-ill group. The AN-rec group had higher levels of body checking than the control group, yet lower than AN-ill. These findings were partly in accordance with our hypotheses as it was expected to find similar levels of body checking for both AN-ill and AN-rec [4]. In the present study, levels of body checking were significantly different in all groups.

#### Body avoiding

In accordance with our hypothesis, groups significantly differed in levels of body avoiding with a large effect size (see Table 1). Levels of body avoiding were significantly different in all groups; reported levels of body avoiding were highest in the AN-ill group, followed by the AN-rec group and lowest in the control group. The planned contrast analysis for body avoiding showed a significant result between the groups as well (t (76.624) = − 18.731, *p* < 0.001) and confirmed group differences.

## IU

Groups significantly differed in levels of IU based on scores of the IUS-12 with a large effect size and confirmed our hypothesis (Table 1). The planned contrast analysis for IU showed a significant result between the groups as well (t (108.616) = − 14.972, *p* < 0.001) and confirmed group differences. The reported levels of IU were highest in the AN-ill group. The AN-rec group had higher levels of IU than the control group, yet lower than the AN-ill group.

### Moderation analyses

**Fig. 1:**
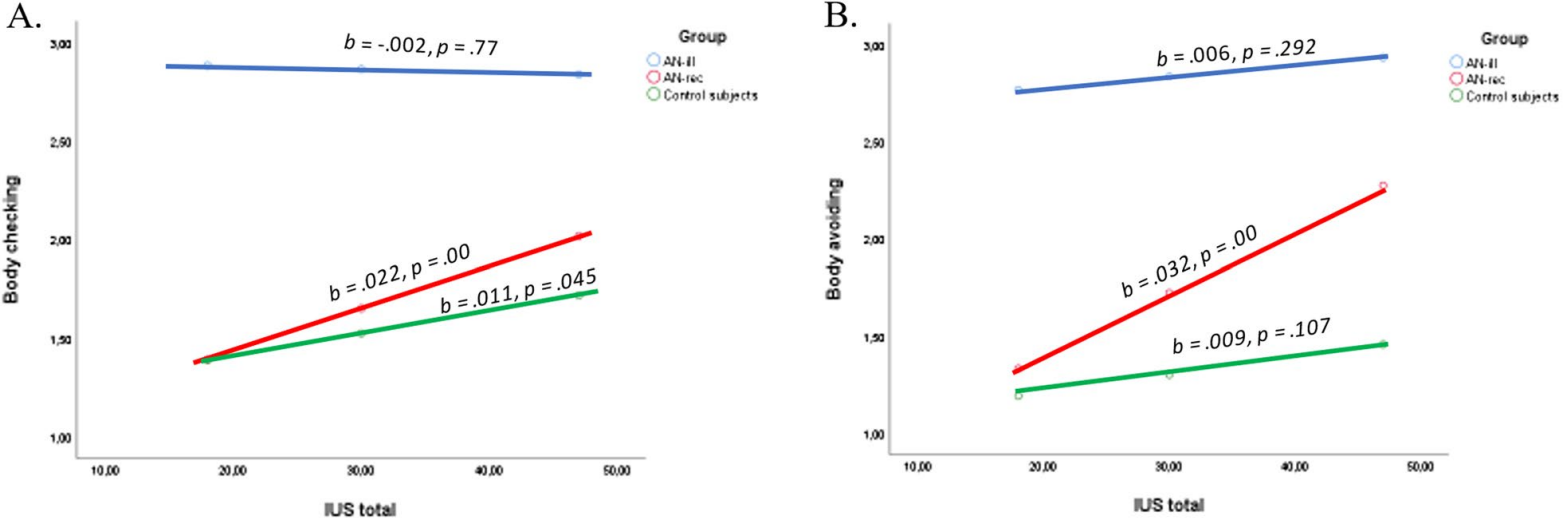
Simple sloples for probing the interactions between IU total and group when predicting body checking and body avoiding. *Note*: IUS total = intolerance of uncertainty. Unstandardized coefficients and p-values are provided to illustrate the direction and the significance of the relationships between independent variables on the x-axis (IU total) and the dependent variables on the y-axis (body checking and body avoiding) in each of the three groups

The group x IU was significant when predicting body checking (*R*^2^ change = 0.014, *F*_(2, 276)_ = 4.88,* p* < 0.01). Higher levels of IU related to body checking in both the AN-rec group and the control group, while the association was nonsignificant in the AN-ill group (Fig. 1a).

The group x IU was significant when predicting body avoiding (*R*^2^ change = 0.018, *F*_(2, 276)_ = 7.76,* p* < 0.01). Higher levels of IU related to body avoiding in the AN-rec group and in the control group, while the association was marginally significant in the control group and nonsignificant within the AN-ill group (Fig. 1b).

## Discussion

To our knowledge, this was the first study investigating IU, body checking, and body avoiding in AN-ill, AN-rec and a control group. Similar to previous findings (e.g., [4, 38, 45, 46, 60], it was found that levels of IU and body avoiding were highest in AN-ill, followed by AN-rec and subsequently in the control group. In contrast to our hypothesis and previous findings [4] where it was expected to find similar levels in both AN-ill and AN-rec, levels of body checking were highest in AN-ill followed by AN-rec and then followed by the control group.

The second part of this study examined whether IU was positively associated with body checking and body avoiding in the three different groups. Regarding the AN-ill group, in contrast to the hypotheses, there were no associations between IU, body checking and body avoiding. One explanation may be that there was a ceiling effect, with the AN-ill group reporting strongly elevated levels of IU, body checking and body avoiding. The ceiling effect is a limitation that occurs when the highest possible score or close to the highest score on a measurement instrument is reached (Salkind [52]), which makes it impossible to reliably estimate the strength of potential associations.

Another explanation for the absence of these associations in AN- ill may be associations of IU with body checking and body avoiding differ across different stages of the illness. Indeed, it has been suggested that body checking and body avoiding may serve a safety behaviour when coping with uncertainty and ameliorate anxiety [44, 64, 67]. Thus, associations of IU with body checking and body avoiding are possibly most salient at the early stage of the disorder, when these behaviours still function as safety behaviours in order to ameliorate anxiety and uncertainty. Possibly, at this stage, eating disorder symptoms may result in behaviours such as restrictive food intake, counting calories but also body checking, and body avoiding and may be initiated to achieve a specific goal. These behaviours may be experienced as reinforcing as they are positively rewarding [61] and, as such, serve the purpose of safety behaviours when attempting to cope with IU and uncertainties in life. In further developed stages of AN, these behaviours are often based on daily repetition and may gradually turn into obsessions and through mechanisms such as reinforcement learning [28], 66. Body checking and body avoiding may ultimately develop into habitual patterns in AN [16] and potentially act as a maintaining factor. At this stage, body checking and body avoiding may no longer be serving as a strategy or safety behaviour to reduce IU, regardless of the higher reported levels of the studied variables in AN-ill. From a clinical perspective, considering body checking and body avoiding as habitual behaviours has implications for understanding mechanisms of this devastating illness and may require different interventions to achieve lasting change [63]. Steinglass et al. [56] reported that techniques from habitual reversal therapy significantly reduced eating disorder symptoms and self-reported habit strengths. Future research should translate these findings on habit forming in AN into interventions that target body checking and body avoiding as well.

Interestingly, within the AN-rec group associations higher levels of IU and more body checking and body avoiding were confirmed. One potential explanation might be that the recovered group is likely to still have some residual symptoms contributing to anxiety. One could also suggest that relations between IU and body checking and body avoiding in those recovered from AN stem from the higher levels of trait anxiety found in individuals with AN that are present prior the onset of the eating disorder and to some extent persist after recovery [37, 47]. Traits refer to stable patterns of behavioural thoughts and emotions over a longer period. Indeed, literature demonstrates a higher prevalence of anxiety-related personality traits in both individuals with AN as well as in those recovered from AN [10, 14, 34, 47]. One could speculate that in individuals who have recovered from AN, treatment might have helped them breaking through this pattern of habits that may have developed during the first stages of the illness by frequently using these safety behaviours such as body checking and body avoiding. Furthermore, during treatment individuals who have recovered from AN may have learnt other ways of coping with trait anxiety and uncertainty and may thus prevent them from relapse. However, traces of trait anxiety are still noticeable within individuals who have recovered. The associations between IU and body checking and body avoiding as found in this study could be considered a remainder but also a vulnerability factor of AN. Therefore, distinguishing between state and trait factors in eating disorders is important for informing etiological, prevention, and treatment models [22] and should be considered carefully within relapse prevention plans.

Lastly, the control group was fully screened for any indication of symptoms of eating pathology. The screening was based on scores on the EDE-Q and BMI among other things [6]. Seeing the low levels of both IU and behaviours, and the lack of any associations between IU and body checking and body avoiding in the control group, findings confirm that for individuals without ED pathology, IU and the behavioural component of body image disturbances may play a role of less importance.

### Strengths and limitations

This study was novel in exploring associations between IU and body checking and body avoiding in three different and distinct groups. Furthermore, this research contributes to an understudied but clinically important topic within body image and AN literature. Our results add to previous findings on body checking and body avoiding and build on an increased understanding of the anxiolytic function of the behavioural component of body image disturbances in AN. However, due to the cross-sectional design, direction of causality in the associations between constructs could not be determined. Longitudinal studies may show patterns of IU and body checking and avoiding over time and would potentially provide a better insight in the development of nonclinical to pathological body image disturbances. Additionally, while this study provided a clear description on how recovery was defined, it should be acknowledged that the definition of recovery is still a continuing debate. Results of a study by Dawson and colleagues [17] suggest that aspects of recovery remain difficult to define and it may be that using different criteria would have led to differing results. Furthermore, future studies might focus on examining all body image components, including the perceptual (e.g., Keizer et al. [35]). It refers to the accuracy of evaluating the own perceived body size and shape relative to the actual body size and shape [29]. In individuals without any symptoms of ED pathology, IU and body checking and body avoiding do not limit daily functioning at all. A better understanding of the interaction between all body image disturbances and their relationship with IU might provide a useful insight in distinguishing and identifying clinical and nonclinical groups, e.g., during a trajectory of recovery, weight gain is pivotal. However, it may lead to increase of uncertainty related to the body that may become intolerable. Addressing IU in relation to these body image disturbances may be a fruitful step forward regarding developing new and additional treatment perspectives.

Another limitation is that data were exclusively based on self-report. In a study by [54], it was discussed that self-assessed recovery might be more subjective and less valid. Furthermore, patients with ED tend to underestimate the severity of their ED (Vandereycken & Van Humbeeck [62]), highlighting the importance of carefully classifying the studied groups. Although we strictly based this study on the definition of recovery derived from the criteria proposed by Bardone-Cone et al. [6], we used the global EDE-Q score to account for the eating disorder cognitions instead of the cut-off scores for the four subscales. Here, we followed the study of Aardoom et al. [1],it showed that the EDE-Q global score was highly accurate in discriminating between individuals with an eating disorder and those without.

### Future studies

In sum, future studies may focus on the course of IU and body checking and body avoiding in relation to the onset, duration and recovery of AN. At different stages of the illness, these constructs might be presented in other ways and may require different approaches with respect to treatment. Studies should preferably include patients with a very short duration of illness to understand the transitional process of habituation of the behavioural patterns related to body image disturbances. Lastly, research should focus on all components of body image disturbances, not solely the cognitive affective and the behavioural, but include the perceptual as well as it could be suggested that it will provide meaningful information on the complexity of these disturbances.

## Conclusions

The present findings contribute to a better understanding of the complexity and dynamical character of body image disturbances. Interestingly, the results of the current study reflected in the differences found between the three studied groups indicate that the associations between IU and body checking and body avoiding may represent different stages of the illness in the clinical groups. The findings of the present study may suggest that during the first stages of AN, body checking and body avoiding may indeed hold the function of safety behaviour aiming to reduce anxiety whilst later in the illness these body related behaviours might gradually transform into a habitual pattern. Future research may study habit forming in relation to body checking and avoiding. Additionally, in the AN-rec group the link between IU and body checking and body avoiding may potentially stem from higher levels of trait anxiety, only now individuals who have recovered from AN know how to cope with these feelings of anxiety and uncertainty in relation to their bodies. Addressing IU in relation to these body image disturbances may be a fruitful step forward regarding developing new treatment perspectives.

## Data Availability

The datasets used and/or analysed during the current study are available from the corresponding author on reasonable request.

## Abbreviations

AN: Anorexia nervosa
AN-rec: Anorexia nervosa recovered
AN-ill: Anorexia nervosa currently ill
APA: American psychiatric association
BCAQ: Body checking and avoidance questionnaire
BMI: Body mass index
DSM-5: Diagnostic and statistical manual of mental disorders
EDE: Eating disorder examination
IU: Intolerance of uncertainty
IUS-12: Intolerance of uncertainty scale
